# Supplementary material for: Hospitalization and mortality associated with SARS-CoV-2 viral clades in COVID-19
Source: Sci Rep. 2021 Feb 26;11:4802. doi: 10.1038/s41598-021-82850-9 (PMC7910290; doi:10.1038/s41598-021-82850-9)
Supplement: Supplementary file 1 — Supplementary Figure. [file 41598_2021_82850_MOESM1_ESM.docx]

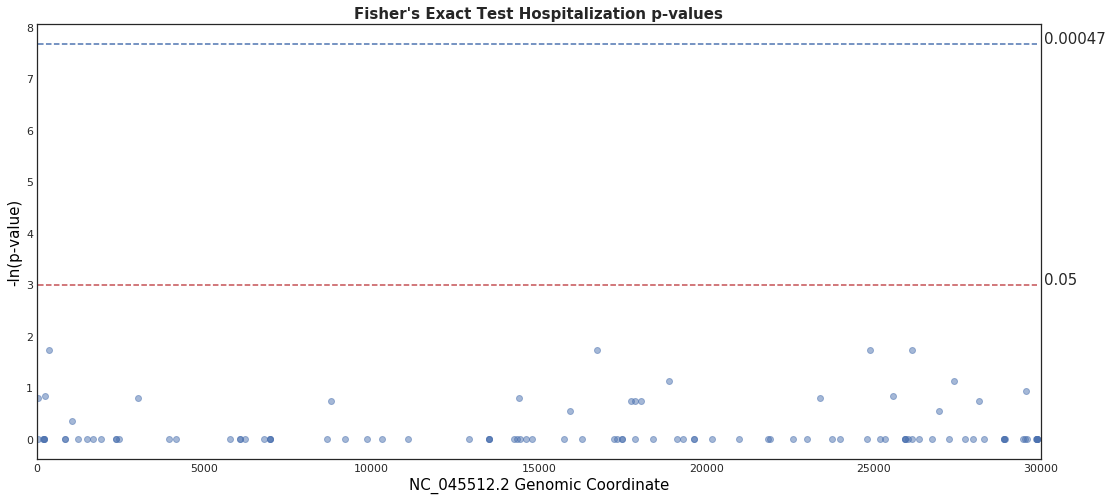


Supplemental Figure 1. Plot of negative logarithm of Fisher’s exact test probability of association with hospitalization for each variant.
